# Pinning Control for the p53-Mdm2 Network Dynamics Regulated by p14ARF

**DOI:** 10.3389/fphys.2020.00976

**Published:** 2020-08-28

**Authors:** Oscar J. Suarez, Carlos J. Vega, Edgar N. Sanchez, Ana E. González-Santiago, Otoniel Rodríguez-Jorge, Alma Y. Alanis, Guanrong Chen, Esteban A. Hernandez-Vargas

**Affiliations:** ^1^Electrical Engineering Department, Centro de Investigación y de Estudios Avanzados del Instituto Politécnico Nacional, Guadalajara, Mexico; ^2^Biomedical Sciences Department, Centro de Investigación Multidisciplinario en Salud, Universidad de Guadalajara, Tonalá, Mexico; ^3^Biochemistry and Molecular Biology Department, Instituto de Investigaciones Básicas y Aplicadas, Universidad Autónoma del Estado de Morelos, Cuernavaca, Mexico; ^4^Computer Sciences Department, Universidad de Guadalajara, Guadalajara, Mexico; ^5^Electrical Engineering Department, City University of Hong Kong, Hong Kong, China; ^6^Frankfurt Institute for Advanced Studies, Frankfurt, Germany

**Keywords:** p53, Mdm2, p14ARF, pinning control, computational modeling

## Abstract

p53 regulates the cellular response to genotoxic damage and prevents carcinogenic events. Theoretical and experimental studies state that the p53-Mdm2 network constitutes the core module of regulatory interactions activated by cellular stress induced by a variety of signaling pathways. In this paper, a strategy to control the p53-Mdm2 network regulated by p14ARF is developed, based on the pinning control technique, which consists into applying local feedback controllers to a small number of nodes (pinned ones) in the network. Pinned nodes are selected on the basis of their importance level in a topological hierarchy, their degree of connectivity within the network, and the biological role they perform. In this paper, two cases are considered. For the first case, the oscillatory pattern under gamma-radiation is recovered; afterward, as the second case, increased expression of p53 level is taken into account. For both cases, the control law is applied to p14ARF (pinned node based on a virtual leader methodology), and overexpressed Mdm2-mediated p53 degradation condition is considered as carcinogenic initial behavior. The approach in this paper uses a computational algorithm, which opens an alternative path to understand the cellular responses to stress, doing it possible to model and control the gene regulatory network dynamics in two different biological contexts. As the main result of the proposed control technique, the two mentioned desired behaviors are obtained.

## 1. Introduction

Gene regulatory networks play key roles in every process of life, including cell cycle, metabolism, signal transduction, cell communication, and cellular differentiation. These complex biological networks use large amounts of data, necessary for modeling, analyzing, and controlling. Mathematical and computational methods are very helpful approaches for constructing network models at molecule level to predict cell behavior under normal conditions or pathological ones. Network topology and interactions between nodes (representing molecules, proteins, genes, mRNA, and others), and edges (establishing regulatory properties) describe the network dynamical behavior (Bolouri and Davidson, 2002). Different mathematical models have been developed for studying gene regulatory networks, which can be divided into four classes (De Jong, 2002): the first ones are logical models, which describe regulatory networks qualitatively, namely, Boolean networks (Kauffman, 1969; Akutsu et al., 1999; Wang et al., 2012), probabilistic Boolean networks (Shmulevich et al., 2002a,b), Bayesian networks (Friedman et al., 2000; Rau et al., 2010), and Petri nets (Chaouiya, 2007; Karlebach and Shamir, 2008); the second ones are defined by continuous models such as ordinary differential equations (Chen et al., 1999; Szallasi et al., 2006; Cao et al., 2012), and the S-system formalism (Kikuchi et al., 2003; Wang et al., 2010); the third ones are single-molecule level models (Cai et al., 2006; Elf et al., 2007; Selvin and Ha, 2008), which account for interactions among molecules; and the last ones are hybrid models combining different formulations like discrete-time and continuous-time frameworks (Ahmad et al., 2006; Fromentin et al., 2010). The continuous-time approach consists in connecting a group of dependent variables to biochemical reaction kinetics. In this case, it is essential to assume that molecules have constant concentrations with respect to cellular compartments, in which their variations are continuous functions of time (Chen et al., 1999; Szallasi et al., 2006; Cao et al., 2012). This approach is adopted in the present paper.

On the other hand, control theory has rapidly developed for complex networks (Wang and Chen, 2002; Sorrentino et al., 2007; Liu and Barabási, 2016). Recently, research focuses on the important issue of how to incorporate control techniques for biological systems and networks (Nowzari et al., 2016; Vinayagam et al., 2016; Gao et al., 2017; Jiao et al., 2018; Papatsenko et al., 2018; Wang et al., 2019), such as pinning control for gene regulatory networks (Lin et al., 2014; Chen et al., 2016; Yue et al., 2017; Li et al., 2018; Burbano et al., 2019). In Lin et al. (2014), a Boolean network model to reproduce the two-phase dynamics of the p53 network in response to DNA damage is developed. In particular, two types of Boolean attractors are presented; the first one is an apoptosis attractor and the second one is a repair attractor. Based on this model, practical control schemes for steering into the apoptosis attractor in presence of DNA damage by pinning the state of a single node or perturbing the weight of a single link are applied. In Chen et al. (2016), an Autonomous Boolean Control Networks (ABCNs) for designing and analyzing the therapeutic intervention strategies are introduced. An important issue in therapeutic intervention is to design a control sequence steering an ABCN from an undesirable location (implying a diseased condition) to a desirable one (corresponding to a healthy condition). Based on this motivation, pinning control strategy is proposed for steering an ABCN from any given condition to the desired one in the shortest time. Cluster synchronization of the coupled genetic regulatory network, represent with ABCN model is investigated in Yue et al. (2017) with a directed topology and using the event-based strategy and pinning control; for this network, a synthetic regulatory network analogous to that in *Escherichia coli* is proposed with twelve states, where three states are the pinned nodes. In Li et al. (2018), a single-input pinning controller design for reachability of Boolean networks is proposed; in addition, different nodes are selected as the pinning ones by solving logical matrix equations and *Drosophila melanogaster* gene regulatory network is used to illustrate the effectiveness and feasibility of the developed method. Finally, Burbano et al. (2019) pinning controllability analysis of multiagent networks subjected to three different types of noise diffusion processes; namely, noise affecting the node dynamics, the communication links, and the pinning control action is done, the effectiveness of the theoretical results is illustrated in the genetic Toggle Switch, originally introduced in *E. coli*. This last publication uses a model based on stochastic differential equations.

Additionally, gene regulatory networks present responses to DNA damage such as cell cycle arrest, DNA repair, senescence, apoptosis among others. Among the main regulators of these responses, tumor suppressor protein p53 (*TP*53) has been recognized as the “guardian of the genome” and is a key component of cellular responses to genotoxic stress (Lane, 1992). p53 regulates the cellular response to genotoxic damage and prevents tumorigenesis by post-translational modifications and gene transactivation (Ashcroft et al., 2000). Without cellular stress, p53 remains inactive and latent due to targeted degradation by the protein E3 ubiquitin-protein ligase Mdm2 (from *MDM*2 proto-oncogene). Mdm2 binds to p53 and marks it for proteasome degradation, preventing p53 accumulation in the nucleus and its transcriptional activity (Momand et al., 1992). In this way, the activity of p53 and its negative regulation by Mdm2 are widely recognized as one of the main regulatory mechanisms in genotoxic stress response (Sionov and Haupt, 1999). The p53-Mdm2 network models have been studied in Wagner et al. (2005), Geva-Zatorsky et al. (2006), Sykes et al. (2006), Wee et al. (2009), Wee et al. (2012), and Hafner et al. (2017), which describe different patterns of response as promotion of transcriptional activities, post-translational modifications, component interactions, and degradation rates. Another important regulator of the p53-Mdm2 network is the tumor suppressor p14ARF (Alternate Reading Frame), one product of the *CDKN*2*A* gene. p14ARF inhibits Mdm2-dependent p53 degradation, through Mdm2-p14ARF complex formation (Zhang et al., 1998). Thus, in response to genotoxic stress induced by gamma-radiation, p14ARF binds directly to Mdm2, leading to an inhibition of Mdm2-mediated p53 ubiquitination and degradation, which increases p53 levels. These events are coupled with downstream signaling pathways, promoting behaviors such as cell cycle arrest, DNA repair, senescence, or apoptosis induction (Zhang et al., 1998; Sionov and Haupt, 1999; Parisi et al., 2002).

The present paper uses a continuous-time approach in a deterministic model of the p53-Mdm2 network regulated by p14ARF under gamma-radiation response (Leenders and Tuszynski, 2013). Pinning control (Li et al., 2004; Chen, 2017) is applied to regulate feedback-loop caused by Mdm2 overexpression stimuli as carcinogenic initial condition (Oliner et al., 1992; Nilbert et al., 1994; Dei Tos et al., 2000; Rayburn et al., 2005), considering no influence of potential mutations. Two desired behaviors are expected; the first one, restoration of an oscillatory pattern, and the second one, the achievement of an increased p53 level expression. p14ARF as pinned node is selected according to the virtual leader methodology (Ren and Beard, 2008; Lewis et al., 2013).

The novelty of the present paper is summarized as follows:

1. The p53 and Mdm2 proteins are controlled by regulating the p14ARF level, based on sensitivity analysis (Dickinson and Gelinas, 1976; Hamby, 1994) as done below.
2. Spanning tree (Ren and Beard, 2008; Lewis et al., 2013) of the p53-Mdm2 network regulated by p14ARF is obtained in order to select the pinned nodes, which are determined based on the virtual leader methodology (Ren and Beard, 2008; Lewis et al., 2013).
3. Pinning control is introduced using a control systems approach where the control dynamics is solved in conjunction with the systems dynamics.
4. The proposed pinning control scheme does not require to apply control inputs to all nodes; in this paper, only one node (the pinned one), ensures oscillatory pattern under gamma-radiation and increased expression of p53 levels for the p53-Mdm2 network.

## 2. Methods

### 2.1. Model Description

**Figure 1:**
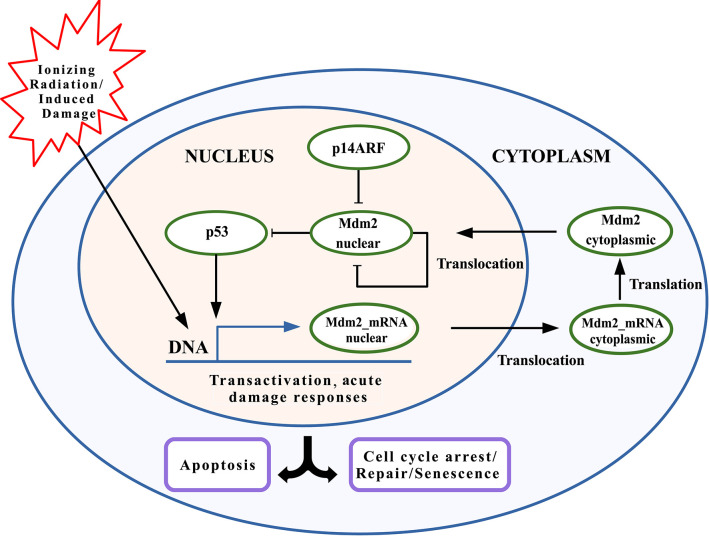
Schematic model of p53 network including Mdm2_*nuclear*_ inhibition by p14ARF. p53 stabilization leads to transcriptional activity, inducing Mdm2_*mRNA*_ expression. Mdm2_*cytoplasmic*_ is transported to the nucleus. p14ARF is also upregulated under genotoxic stress, where binds to Mdm2_*nuclear*_ and causing its suppression. p53 downstreaming possible responses include cell cycle arrest, DNA repair, senescence, and/or apoptosis.

The p53-Mdm2 network is key for determining cell behavior in response to cellular stress, such as DNA damage induced by gamma-radiation (Strigari et al., 2014; Hage-Sleiman et al., 2017), which can start a program of p53-dependent consequences such as cell cycle arrest, DNA repair, senescence, or apoptosis induction (Wagner et al., 2005; Geva-Zatorsky et al., 2006; Sykes et al., 2006; Wee et al., 2009, 2012; Hafner et al., 2017). In Figure 1, the interaction network between p53, Mdm2, and p14ARF is provided for an individual cell model in two cell compartments: nucleus and cytoplasm. This model includes DNA damage induced by gamma-radiation, which generates the p53 activation and the transactivation of both *TP*53 and *MDM*2 (among other transactivated genes, which are not considered in this model). Initially, the translocation process of mRNAs to the cytoplasm and its subsequent translation into proteins takes place; afterward, proteins are transported back to the nucleus. While p53 remains at high levels, Mdm2_*nuclear*_ reduces its concentration levels, and vice versa, thus, producing an oscillatory pattern. Mdm2_*cytoplasmic*_ moves to the nucleus at a constant rate, ignoring all other possible behaviors. The production and degradation rates of p14ARF remain constant (Leenders and Tuszynski, 2013).

Following assumptions for the p53-Mdm2 network regulated by p14ARF are presented:

1. The p53-Mdm2 network is one of the most explored biological mechanisms that exist, which provides adequate information.
2. The biological context requires different scenarios dependent on p53 and failure in the network of p53-Mdm2-p14ARF, which can be represented in equations for simulation output desired reference that have a biological explanation, such as regulation mechanisms in gene expression.
3. The biological responses of p53 that we are interested in exploring are related to the ability to generate cell cycle arrest, DNA repair, senescence, and/or apoptosis, especially the last one since it has a key role in the tumor suppressor response.
4. The model assumes the response to an ionizing radiation stimulus with p53-dependent responses, which presents an oscillatory pattern due to variation between p53 and its inhibitor Mdm2. The main objective is to simulate the p53 suppressed response when Mdm2 is overexpressed, which has been documented in several types of tumors.

### 2.2. Mathematical Description

Taken from Leenders and Tuszynski (2013), based on the principle of mass-action and the saturable transcription kinetics, the p53-Mdm2 network regulated by p14ARF without control action is mathematically described as follows:

(1) $$\begin{array}{l} \frac{d\left[p53\right]}{dt}=k_{p}-k_{1}\left[p53\right]\left[Mdm2_{nuclear}\right]\\ \;-d_{p}\left[p53\right], \end{array}$$

(2) $$\begin{array}{l} \frac{d\left[Mdm2\text{\_}mRNA_{nuclear}\right]}{dt}=k_{m}+k_{2}\frac{{\left[p53\right]}^{1.8}}{{K_{D}}^{1.8}+{\left[p53\right]}^{1.8}}\\ \;-k_{0}\left[Mdm2\text{\_}mRNA_{nuclear}\right], \end{array}$$

(3) $$\begin{array}{l} \frac{d\left[Mdm2\text{\_}mRNA_{cytoplasmic}\right]}{dt}=k_{0}\left[Mdm2\text{\_}mRNA_{nuclear}\right]\\ \;-d_{rc}\left[Mdm2\text{\_}mRNA_{cytoplasmic}\right], \end{array}$$

(4) $$\begin{array}{l} \frac{d\left[Mdm2_{cytoplasmic}\right]}{dt}=k_{T}\left[Mdm2\text{\_}mRNA_{cytoplasmic}\right]\\ \;-k_{i}\left[Mdm2_{cytoplasmic}\right], \end{array}$$

(5) $$\begin{array}{l} \frac{d\left[Mdm2_{nuclear}\right]}{dt}=k_{i}\left[Mdm2_{cytoplasmic}\right]\\ \;-d_{mn}{\left[Mdm2_{nuclear}\right]}^{2}\\ \;-k_{3}\left[Mdm2_{nuclear}\right]\left[p14ARF\right], \end{array}$$

(6) $$\begin{array}{l} \frac{d\left[p14ARF\right]}{dt}=k_{a}-d_{a}\left[p14ARF\right]\\ \;-k_{3}\left[Mdm2_{nuclear}\right]\left[p14ARF\right]. \end{array}$$

The deterministic model (1–6) includes: p53 production and degradation (Equation 1), *Mdm*2_*mRNA*_*nuclear*_ basal transcription (p53-dependent and Mdm2-independent production) in Equation (2); *Mdm*2_*mRNA*_*cytoplasmic*_ transport from nucleus to cytoplasm (Equation 3), *Mdm*2_*mRNA*_*cytoplasmic*_ translation rate and Mdm2_*cytoplasmic*_ protein transport to nucleus (Equation 4), Mdm2_*cytoplasmic*_ decay through Mdm2_*nuclear*_-p14ARF complex, which removes Mdm2 and stops Mdm2_*nuclear*_ ubiquitination rate (Equation 5), p14ARF production and p14ARF decay in nucleus compartment (Equation 6). The parameter values used are presented in Table 1.

**Table 1:** Model parameters.

**Parameter**	**Description**	**Value**	**References**
*k*_*p*_	p53 production	0.5 proteins/s	Leenders and Tuszynski, 2013
*k*_1_	Mdm2-dependent p53 degradation	9.963 × 10^−6^/s	Leenders and Tuszynski, 2013
*d*_*p*_	p53 decay	1.925 × 10^−5^/s	Leenders and Tuszynski, 2013
*k*_*m*_	p53-independent Mdm2 production	1.5 × 10^−3^ RNA/s	Leenders and Tuszynski, 2013
*k*_2_	p53-dependent Mdm2 production	1.5 × 10^−2^/s	Weinberg et al., 2005
*K*_*D*_	Dissociation constant in the promoter region	740 proteins	Weinberg et al., 2005
*k*_0_	RNA transport from nucleus to cytoplasm	8.0 × 10^−4^/s	Leenders and Tuszynski, 2013
*d*_*rc*_	Mdm2_mRNA decay in cytoplasm	1.444 × 10^−4^/s	Hsing et al., 2000
*k*_*T*_	Translation rate	1.66 × 10^−2^ proteins/s	Cai and Yuan, 2009
*k*_*i*_	Protein transport from cytoplasm to nucleus	9.0 × 10^−4^/s	Mor et al., 2010
*d*_*mn*_	Mdm2 autoubiquitination	1.66 × 10^−7^/s	Leenders and Tuszynski, 2013
*k*_3_	Mdm2_*nuclear*_-p14ARF complex formation rate	9.963 × 10^−6^/s	Northrup and Erickson, 1992
*K*_*a*_	p14ARF production	0.5 proteins/s	Leenders and Tuszynski, 2013
*d*_*a*_	p14ARF decay	3.209 × 10^−5^/s	Kuo et al., 2004

#### 2.2.1. Model Characteristics of p53-Mdm2 Network Regulated by p14ARF

- The model (1–6) is based on response to gamma radiation in individual MCF-7 cells. This model does not represent all single cell line type responses to gamma radiation.
- It is important to emphasize that the model employed (1–6) describes the core components of the p53 network and are relevant to determine p53 dynamics in response to gamma radiation-induced DNA damage. Mathematical model considers several parameters with non-linear behaviors such as molecule production, degradation, the dissociation constant in the promoter region, translocation of network components, complex formation rate, and translation rate. Other genes/molecules regulations are ignored, gene mutations are not considered, and constant molecule concentrations in the cell are assumed.
- Note that this model includes both experimentally measured and unknown parameters, which are selected manually in order to fit the oscillatory behavior observed for one cell model, as reported in Leenders and Tuszynski (2013).
- Due to the scarcity of parameters used in equations, implications in future researches will involve eliciting responses to stimuli such as gamma radiation, also in other stimuli with a p53-dependent and independent responses, in single cells and multiple ones, including detailed characterizations about genome integrity so that a mathematical model reaches excellent precision.

### 2.3. Pinning Control Methodology

Consider a general network consisting of *N* nodes with nonlinear couplings, where each node is a scalar nonlinear dynamical system, which represents genes, concentrations of RNAs and proteins, given by

(7) $$\begin{array}{l} {\dot{x}}_{i}=f_{i}\left(x_{i}\right)+h_{i}\left(t,x_{1},x_{2},\ldots ,x_{N}\right),\;i=1,2,\ldots ,N, \end{array}$$

where *x*_*i*_ ∈ ℝ is the state of node *i*, for *i* = 1, 2, …, *N*; *f*_*i*_:ℝ↦ℝ represents the self-dynamics of node *i* related to the degradation process of RNA and proteins, and so on; $h_{i}:{\mathbb{R}}^{N}\mapsto \mathbb{R}$ is the nonlinear coupling function between nodes, associated with the changes of *x*_*i*_ due to transcription, translation, repression, activation, or other interaction processes, and *N* represents the all network nodes.

The control objective is that (7) tracks a desired output trajectory, given by

$$y=y_{r}\left(t\right).$$

To achieve this objective, local feedback controllers are applied to a reduced number of network nodes, according to the pinning control methodology (Wang and Chen, 2002; Li et al., 2004; Chen, 2017). This methodology is composed of two parts: the first one, pinned nodes *l* are selected to apply control actions as in (8), where 1 ≤ *l* ≤ *N*, and *l* can be as small as one. The second one is the remained network nodes (*N* − *l*) without control action as in (9). Thus, the controlled network can be written as

(8) $$\begin{array}{l} {\dot{x}}_{i}=f_{i}\left(x_{i}\right)+h_{i}\left(t,x_{1},x_{2},\ldots ,x_{N}\right)+g_{i}\left(x_{i}\right)u_{i},\;i=1,2,\ldots ,l. \end{array}$$

(9) $$\begin{array}{l} {\dot{x}}_{i}=f_{i}\left(x_{i}\right)+h_{i}\left(t,x_{1},x_{2},\ldots ,x_{N}\right),\;i=l+1,l+2,\ldots ,N. \end{array}$$

where *g*_*i*_:ℝ↦ℝ is a nonlinear function of the node state *i*, for *i* = 1, 2, …, *l* and *u*_*i*_ denotes the control on the node *i* ∈ *l*.

In the present paper, *u*_*i*_ in (8), is proposed as a local positive discontinuous feedback control law, described by

(10) $$\begin{array}{l} u_{i}=\{\begin{array}{cc} 1+K_{i}\left(1-e_{i}\right), & \text{if }|\varphi_{i}|<1,\\ 1+K_{i}\left(1-sign\left(e_{i}\right)\right), & \text{if }|\varphi_{i}|>1, \end{array} \end{array}$$

where *K*_*i*_ is a positive control gain selected by the designer, *e*_*i*_ is the tracking error between the desired output trajectory [*y*_*r*_(*t*)] and the controlled state (*x*_*i*_), is given by

(11) $$\begin{array}{l} e_{i}=\left(x_{i}-y_{r}\left(t\right)\right), \end{array}$$

with $\varphi_{i}=\frac{e_{i}}{S_{i}}$ being a proposed auxiliary variable to reject chattering effect caused by *sign*(·) (signum function extracts the sign of a real number) (), and *S*_*i*_ a signal filter given by

(12) $$\begin{array}{l} {\dot{S}}_{i}=-\alpha_{i}S_{i}+\omega_{i},\;i=1,2,\ldots ,l, \end{array}$$

where α_*i*_ and ω_*i*_ are positive gains to be selected.

### 2.4. p14ARF as Pinned Node

**Figure 2:**
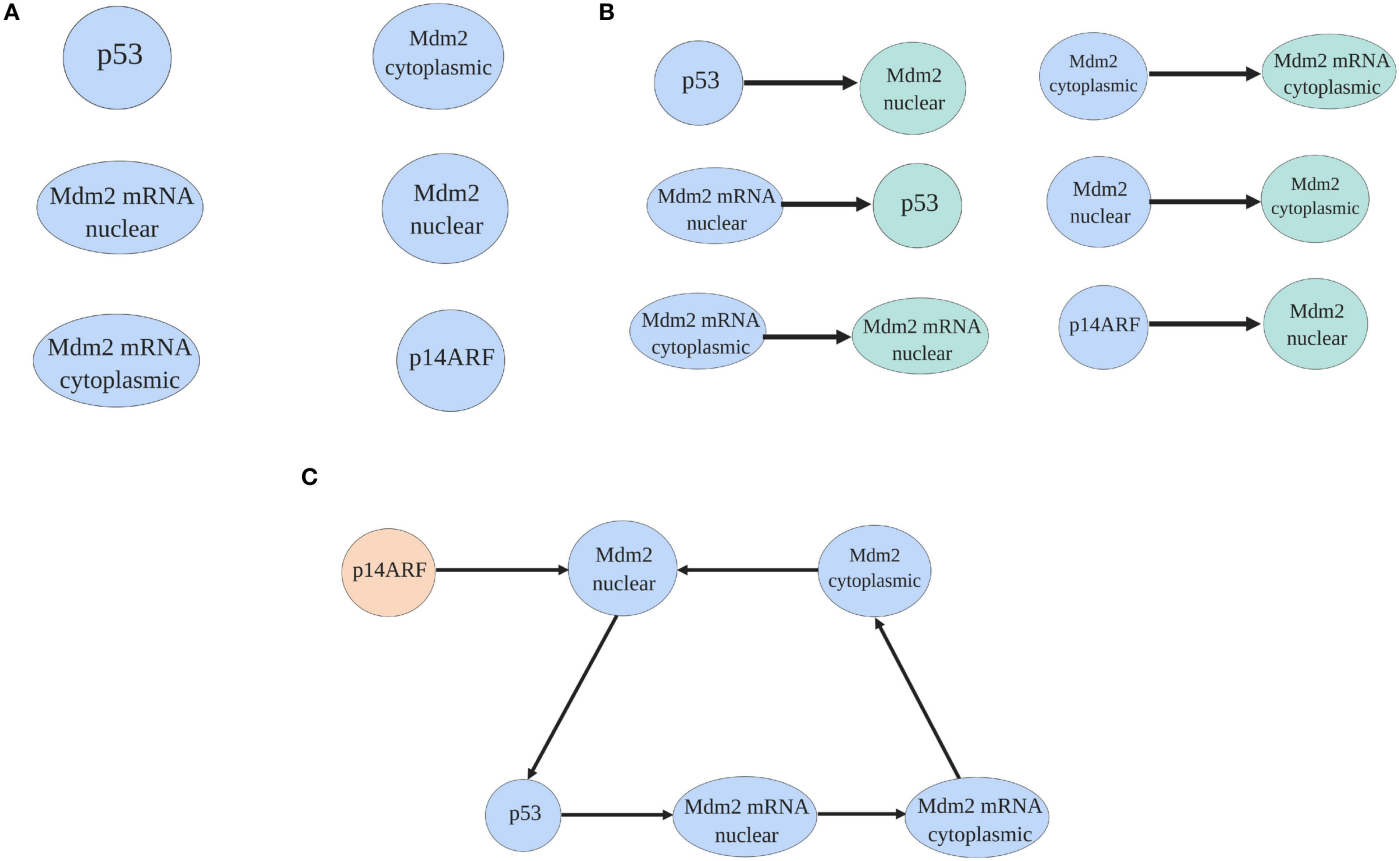
Spanning tree process of the p53-Mdm2 network regulated by p14ARF. In **(A)** proteins and mRNA of the network are presented. In **(B)** based on the mathematical model (1–6) and doing a biological analysis, proteins, and mRNA are illustrated with their dependent molecule. Finally, in **(C)** the spanning tree where p14ARF do not have direct dependence (virtual leader) from other protein/mRNA is presented.

To select the pinned nodes, the virtual leader methodology presented in Ren and Beard (2008) and Lewis et al. (2013) is used. The methodology consists on analyzing the interactions between proteins presented in Figure 1 using the mathematical model (1–6). The nodes that affect directly or indirectly the dynamical behavior everyone else, are candidates as pinned nodes. In this sense, the spanning tree of the p53-Mdm2 network regulated by p14ARF is developed, as in Figure 2; based on this analysis, p14ARF is the adequate biological selection as the pinned node.

From Equation (6), the differential equation for p14ARF (pinned node) is defined by three cellular processes, as follows

$$\begin{array}{l} \frac{d\left[p14ARF\right]}{dt}=\overset{\text{Production}}{\overset{︷}{k_{a}}}\;\overset{\text{Degradation}}{\overset{︷}{-d_{a}\left[p14ARF\right]}}\\ \overset{Mdm2_{nuclear}-p14ARF\text{ complex formation}}{\overset{︷}{-k_{3}\left[Mdm2_{nuclear}\right]\left[p14ARF\right]}}. \end{array}$$

In order to control the p53-Mdm2 network, it is necessary to increase the p14ARF concentration levels to regulated Mdm2_*nuclear*_ production. As can be seen degradation process and *Mdm*2_*nuclear*_ − *p*14*ARF* complex formation have a negative sign; while, the production process has a positive sign. Due to this fact, we propose to modify the p14ARF production (*K*_*a*_) process added the control law (10).

Thus, considering Equations (8) and (9), the p53-Mdm2 network regulated by p14ARF with control action is mathematically described as follows:

(13) $$\begin{array}{l} \frac{d\left[p14ARF\right]}{dt}=k_{a}u_{1}-d_{a}\left[p14ARF\right]\\ \;-k_{3}\left[Mdm2_{nuclear}\right]\left[p14ARF\right], \end{array}$$

(14) $$\begin{array}{l} \frac{d\left[p53\right]}{dt}=k_{p}-k_{1}\left[p53\right]\left[Mdm2_{nuclear}\right]\\ \;-d_{p}\left[p53\right], \end{array}$$

(15) $$\begin{array}{l} \frac{d\left[Mdm2\text{\_}mRNA_{nuclear}\right]}{dt}=k_{m}+k_{2}\frac{{\left[p53\right]}^{1.8}}{{K_{D}}^{1.8}+{\left[p53\right]}^{1.8}}\\ \;-k_{0}\left[Mdm2\text{\_}mRNA_{nuclear}\right], \end{array}$$

(16) $$\begin{array}{l} \frac{d\left[Mdm2\text{\_}mRNA_{cytoplasmic}\right]}{dt}=k_{0}\left[Mdm2\text{\_}mRNA_{nuclear}\right]\\ \;-d_{rc}\left[Mdm2\text{\_}mRNA_{cytoplasmic}\right], \end{array}$$

(17) $$\begin{array}{l} \frac{d\left[Mdm2_{cytoplasmic}\right]}{dt}=k_{T}\left[Mdm2\text{\_}mRNA_{cytoplasmic}\right]\\ \;-k_{i}\left[Mdm2_{cytoplasmic}\right], \end{array}$$

(18) $$\begin{array}{l} \frac{d\left[Mdm2_{nuclear}\right]}{dt}=k_{i}\left[Mdm2_{cytoplasmic}\right]\\ \;-d_{mn}{\left[Mdm2_{nuclear}\right]}^{2}\\ \;-k_{3}\left[Mdm2_{nuclear}\right]\left[p14ARF\right]. \end{array}$$

The p53-Mdm2 network regulated by p14ARF without control action (1–6) and with control action (13–18) are simulated using Matlab/Simulink and the fourth-order Runge-Kutta integration method with a fixed step size of 1 × 10^−3^.

## 3. Results

### 3.1. Behaviors of the p53-Mdm2 Network Regulated by p14ARF Without Control Action

**Figure 3:**
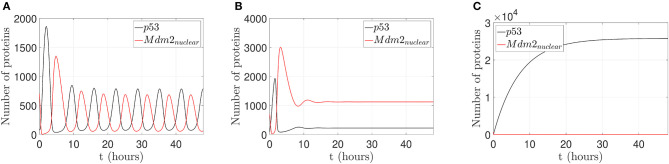
Behaviors of the p53-Mdm2 network regulated by p14ARF without control action. **(A)** p53-Mdm2_*nuclear*_ oscillatory pattern under gamma-radiation within a lapse of 6.64 h. **(B)** Mdm2_*nuclear*_ overexpression and p53 downregulation with $k_{1}=1.9926\times 10^{-6}$/s and $k_{2}=45\times 10^{-3}$/s; once Mdm2_*nuclear*_ is overexpressed, p53 is inhibited. It can be conjectured that Mdm2_*nuclear*_ deregulation will lead to oncogenic behavior through p53 suppression. **(C)** Increased expression of p53 levels response under gamma-radiation induction with *k*_*a*_ = 5 proteins/s and $d_{rc}=1.444\times 10^{-2}$/s. It can be conjectured that p53 upregulation will lead to cell cycle arrest, DNA repair, senescence, and/or apoptotic response.

Three different behaviors of the p53-Mdm2 network without control actions are presented in Figure 3.

As displayed in Figure 3A, the p53-Mdm2 network presents an oscillatory pattern under gamma-radiation, for a lapse of 48 h, using the parameter values shown in Table 1. This response is due to post-translational modifications of p53 and the negative interactive loop of Mdm2-mediated ubiquitination, according to Ciliberto et al. (2005) and Geva-Zatorsky et al. (2006); this pattern has not been observed for all cell types and requires wild-type genes (Lahav et al., 2004; Leenders and Tuszynski, 2013).

Figure 3B illustrates that p53-Mdm2 dependent affinity is altered, producing a Mdm2_*nuclear*_ overexpression when the parameters *k*_1_ and *k*_2_ are set to values five and ten times larger than the original ones, respectively. This behavior is reported in a variety of human soft tissue tumors and in hematological malignancies, as discussed in Oliner et al. (1992), Nilbert et al. (1994), Dei Tos et al. (2000), Bond et al. (2004), and Rayburn et al. (2005). In human tumors, Mdm2 overexpression can inhibit p53 normal regulatory activities and induce a loss of growth-inhibitory signals in cytostatic and apoptotic responses, which may favor a carcinogenic process.

Figure 3C displays an increased expression of p53 levels when the parameters *K*_*a*_ and *d*_*rc*_ are set to values ten and one hundred times larger than the original ones, respectively. This p53 dynamical behavior is related to downstream genes involved in signaling pathway, which can produce cell cycle arrest, DNA repair, senescence, and/or apoptotic response (El-Deiry, 1998; Hsing et al., 2000; Khan et al., 2004; Arya et al., 2010; Purvis et al., 2012).

### 3.2. Behaviors of the p53-Mdm2 Network With Nutlin-3

Several molecular inhibitors of p53-Mdm2 interaction have been proposed, including a small molecule called Nutlin-3 as in Vassilev et al. (2004). Due to Mdm2 deregulation has been reported in various tumor types, Nutlin-3 has been an option to block the p53-binding site of MDM2 competitively and induce the upregulation and activation of p53 pathway (Yee-Lin et al., 2018). These findings motivated us to observe the possible effect of the external stimulus of Nutlin-3 on the oscillatory pattern behavior in the p53-Mdm2 network regulated by p14ARF. If p53 stabilization is achieved and p53 degradation is avoided, as reported for Nutlin-3 in tumors, p53 can accomplish antiproliferative effects.

**Figure 4:**
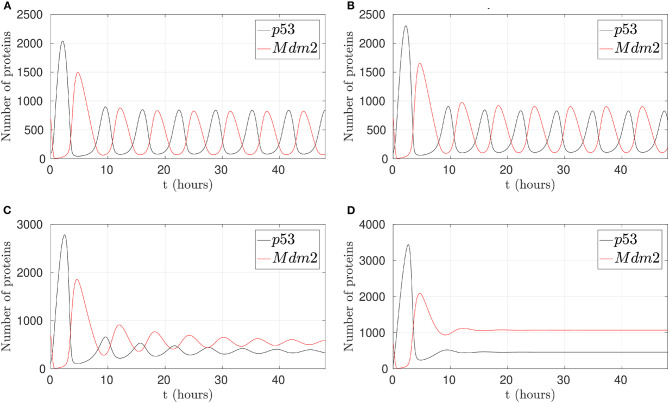
Behaviors of the p53-Mdm2 network regulated by p14ARF under Nutlin-3 effect. **(A)** With 25% activity in k1. **(B)** With 50% activity in k1. **(C)** With 75% activity in k1. **(D)** With 90% activity in k1. **(C,D)** Shows desynchronized p53-Mdm2 interaction.

In this sense, a simulation to reduce p53-Mdm2 interaction and p53 degradation in the presence of Nutlin-3 is presented in Figure 4. To achieve this behavior, k1, which represents Mdm2-dependent p53 degradation, takes values between 25, 50, 75, and 90% less from the original value. It is possible to observe that simulating the effect of Nutlin-3, liberates p53 from the interaction and inhibition by Mdm2 in the network, allowing to desynchronize p53 and MDM2 oscillatory behavior, reaching a stable high concentration when the inhibition by Nutlin-3 is strong.

### 3.3. Sensitivity Analysis for p14ARF Production (*K*_*a*_)

**Figure 5:**
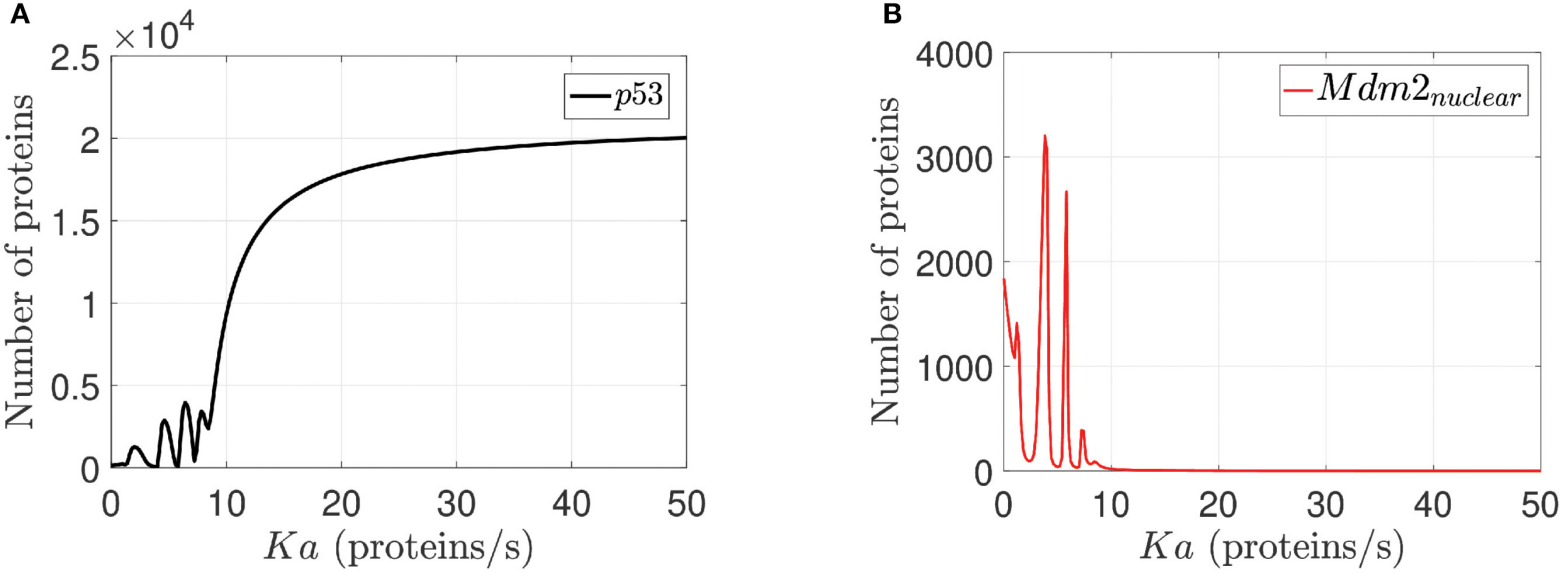
Sensitivity analysis for *K*_*a*_. In **(A)** p53 response to *K*_*a*_ variations is illustrated. In **(B)** Mdm2_*nuclear*_ response to *K*_*a*_ variations is presented. For both cases *K*_*a*_ varies between 0 to 50 *proteins*/*s* for all network proteins, but we select p53 and Mdm2_*nuclear*_ because they are the output desired in the network.

This analysis is done on the basis of p14ARF production (*K*_*a*_) value variation effects on p53 and Mdm2_*nuclear*_ respectively as can be seen in Figure 5. Sensitivity analysis (Dickinson and Gelinas, 1976; Hamby, 1994) determines the *K*_*a*_ values for which the network can not achieve desired behaviors (0–1.5 *proteins*/*s*); the *K*_*a*_ value to reach p53-Mdm2_*nuclear*_ oscillatory pattern (1.6–9.5 *proteins*/*s*), and the *K*_*a*_ value to generate an increased expression of p53 with Mdm2_*nuclear*_ downregulation (9.6–50 *proteins*/*s*) are determined from Figure 5.

### 3.4. Behaviors of the p53-Mdm2 Network Regulated by p14ARF With Control Action

To illustrate the p53 and Mdm2_*nuclear*_ behavior under pinning control actions, two cases are considered: (1) to restore an oscillatory pattern under gamma-radiation, and (2) to achieve an increased expression of p53 level. For a 24 h lapse, the network runs without any control action and presents overexpressed Mdm2-mediated p53 degradation as carcinogenic initial behavior for both cases (Oliner et al., 1992; Nilbert et al., 1994; Dei Tos et al., 2000; Bond et al., 2004; Rayburn et al., 2005), which is displayed in Figure 3B above.

#### 3.4.1. Case 1: Restoration of an Oscillatory Pattern Under Gamma-Radiation

**Figure 6:**
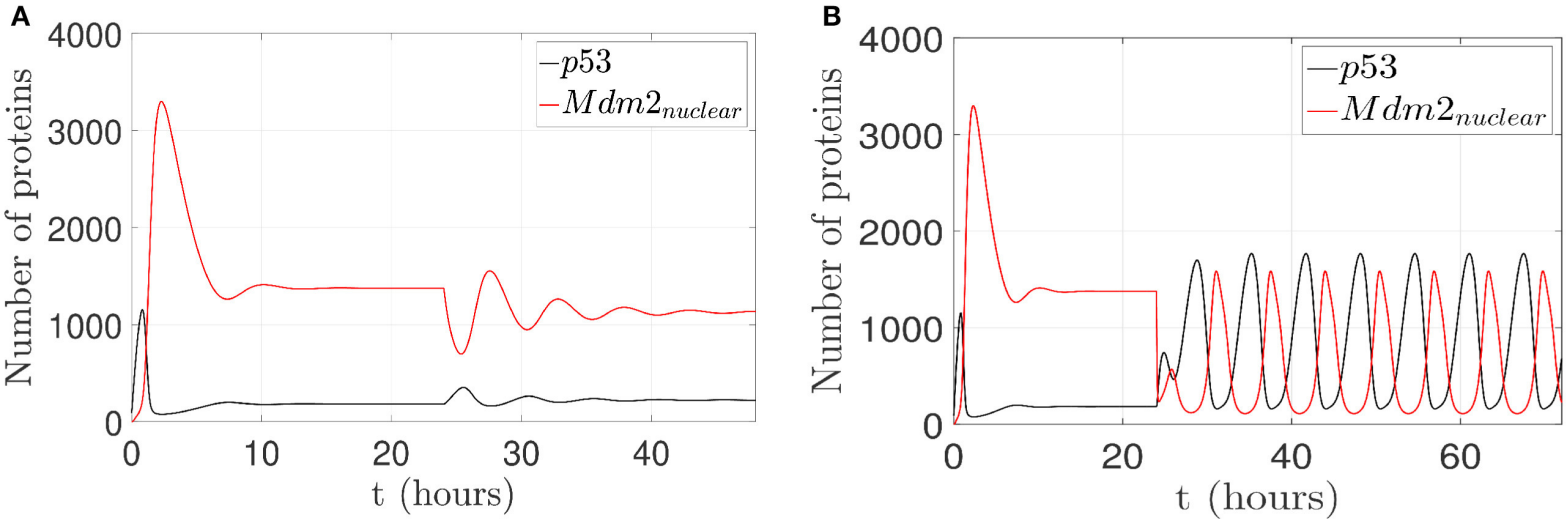
Proposed scenario simulations to achieve restoration of an oscillatory pattern under gamma-radiation in the p53-Mdm2 network regulated by p14ARF. For both tests is possible to observe overexpressed Mdm2-mediated p53 degradation as carcinogenic initial behavior at first 24 h. In **(A)**, control action with (*k*_*a*_*u*_1_) in a range between 0 and 1.5 *proteins*/*s*, the oscillatory pattern under gamma-radiation is not achieved (24–48 h) and likewise in **(B)** oscillatory pattern under gamma-radiation under pinning control (24–72 h) with (*k*_*a*_*u*_1_) in a range between 1.6 and 9.5 *proteins*/*s* is achieved.

For the first case, by considering p14ARF production (*k*_*a*_*u*_1_) in a range between 0 and 1.5 *proteins*/*s*, the proposed controller is turned on after the 24 h initial lapse; however, the network can not achieves the oscillatory pattern as can be seen in Figure 6A. Due to the low value of *k*_*a*_*u*_1_, the pinning control technique cannot achieve the desired behavior. Otherwise, with (*k*_*a*_*u*_1_) in a range between 1.6 and 9.5 *proteins*/*s*, the proposed controller forces the network to gradually track the oscillatory pattern as can be seen in Figure 6B.

#### 3.4.2. Case 2: Achievement of a p53 Level Increased Expression

**Figure 7:**
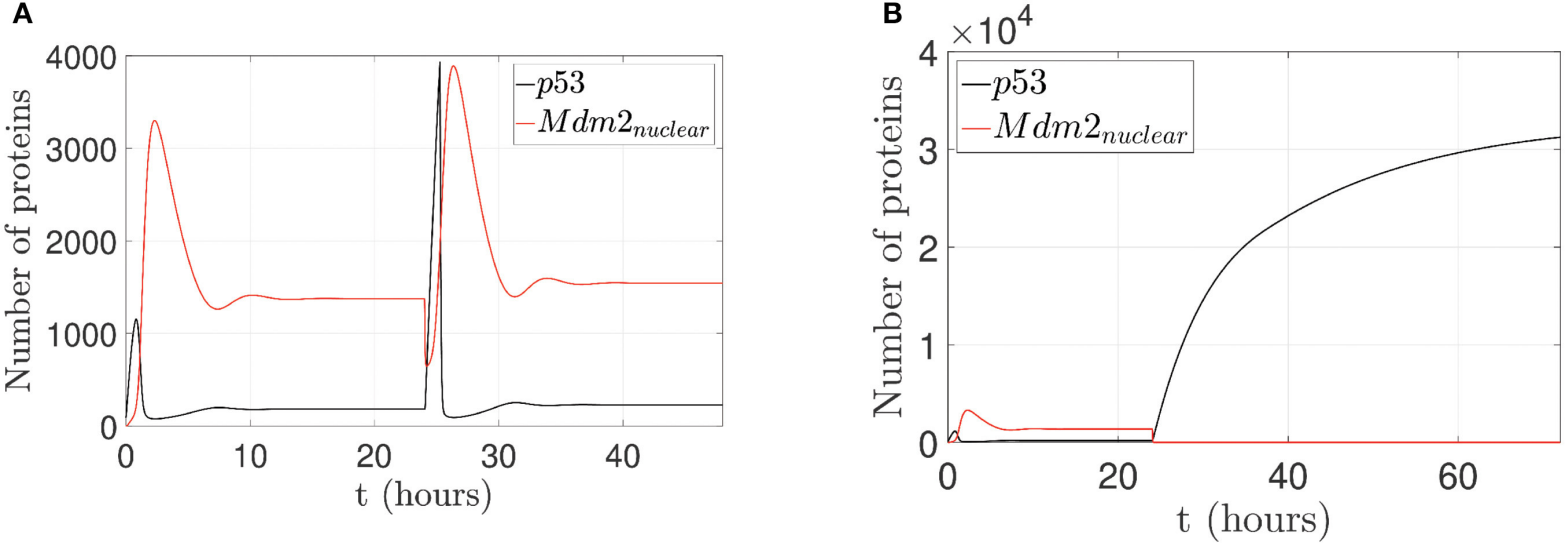
Proposed scenario simulations to achieve p53 level increased expression in the p53-Mdm2 network regulated by p14ARF. For both tests is possible to observe overexpressed Mdm2-mediated p53 degradation as carcinogenic initial behavior at first 24 h. In **(A)**, control action with (*k*_*a*_*u*_1_) in a range between 0 and 1.5 *proteins*/*s*, increased expression of p53 response is not achieved (24–48 h) and likewise in **(B)** increased expression of p53 response under pinning control (24–72 h) with (*k*_*a*_*u*_1_) in a range between 9.6 and 50 *proteins*/*s* is achieved.

For the second case, by considering p14ARF production (*k*_*a*_*u*_1_) in a range between 0 and 1.5 *proteins*/*s*, the proposed controller is turned on; however, the network can not achieves increased expression of p53 as can be seen in Figure 7A. Due to the low value of *k*_*a*_*u*_1_, the pinning control technique cannot achieve the desired behavior. Otherwise, with (*k*_*a*_*u*_1_) in a range between 9.6 and 50 *proteins*/*s*, the proposed controller again is turned on, and the system gradually tracks the increased expression of p53 levels as can be seen in Figure 7B.

**Figure 8:**
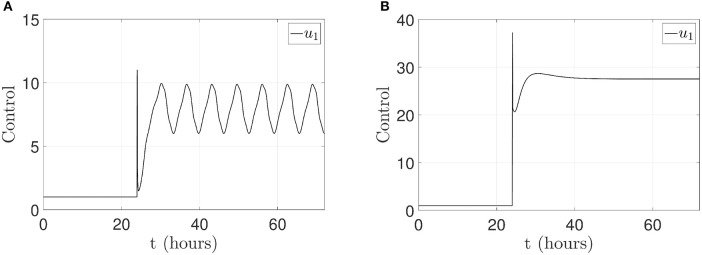
Control signal *u*_1_(*t*) applied in p14ARF. **(A)** Case 1: control action to achieve an oscillatory pattern under gamma-radiation. **(B)** Case 2: control action to achieve an increased expression of p53 response.

For both cases, the control law (*u*_1_(*t*) ∈ ℝ) is applied to p14ARF node as in Equation (14). The respective control actions are displayed in Figure 8A for the first case and Figure 8B for the second case. From the above results, it can be clearly seen that the pinning controller achieves regulation successfully for the p53-Mdm2 network.

## 4. Discussion

In biological systems, gene regulatory networks, having complex interactions, present important challenges for the application of control strategies. A mixture of mechanisms such as gene expression patterns, post-translational modifications, translocation, components degradation, and the specific changes of internal and external signals of the cell, generate nonlinear behavior. Hence, to illustrate the applicability of complex network control, we present two cases for a deterministic network model corresponding to tumor suppressor p53, Mdm2, and p14ARF.

### 4.1. Behaviors Induced Without Control Action

#### 4.1.1. p53-Mdm2_*nuclear*_ Oscillatory Pattern

Levels of p53 and Mdm2_*nuclear*_ proteins present oscillatory behavior, caused by pulses resulting from p53 activation, p53-dependent transactivation, Mdm2 production, Mdm2_*nuclear*_ sequestration by p14ARF, and Mdm2-mediated ubiquitination. This process allows the cell to respond to double-strand breaks (DSBs) in DNA induced by gamma radiation, which correlates with the number of p53 pulses in individual cells (Lahav et al., 2004; Geva-Zatorsky et al., 2006). For the model used in this paper, there are regulatory factors which have not been considered, as the interaction of other potentially relevant genes transactivated by p53 (nearly 100 genes in pathways such as cell cycle arrest, DNA repair, senescence, and apoptosis; Riley et al., 2008). One of the simpler explanations for the p53 oscillatory pattern is due to repeated activation of ATM (Ataxia Telangiectasia Mutant), which dissociates the p53-Mdm2 complex and stabilizes an increase in p53 levels. These changes are driven by persistent DNA damage induced by radiation (Lahav et al., 2004; Geva-Zatorsky et al., 2006). Therefore, recovering the normal pattern response to DNA damage by the p53-Mdm2 network is fundamental for tumor suppression.

#### 4.1.2. Mdm2_*nuclear*_ Overexpression and p53 Downregulation

There are gene abnormalities in tumors, carcinogenesis driven by viral infections, and other mechanisms that contribute to inactivate p53 functions and its signaling outcomes (Scheffner et al., 1990; Camus et al., 2003; Rayburn et al., 2005). For example, Mdm2 overexpression leads to p53 downregulation, contributing to losses of tumor suppressor activity. Mdm2 overexpressed is a hallmark in several types of cancer (Nilbert et al., 1994; Dei Tos et al., 2000; Rayburn et al., 2005). As reviewed in Rayburn et al. (2005), Mdm2 is overexpressed in liposarcomas, osteosarcomas, testicular germ cell tumors, embryonic carcinomas, brain tumors (including glioblastomas and astrocytomas), hematological malignancies, bladder cancer, breast cancer, colorectal cancer among others. In this sense, the number of Mdm2 abnormalities is highly variable. Moreover, not all samples from the same type of tumor, show Mdm2 overexpression. Bond et al. (2004) reported other mechanisms that promote Mdm2 overexpression, where a single nucleotide polymorphism (SNP309) in the MDM2 promoter, increases the affinity of the transcriptional activator Sp1, resulting in higher levels of Mdm2 mRNA and Mdm2 translation rate. This behavior illustrates p53 downregulation resulting in a decreased response to DNA damaging agents and acceleration of tumorigenesis.

#### 4.1.3. Increased Expression of p53 Levels

The stabilization and accumulation of p53 levels are part of the DNA damage response to maintain genome integrity. One of the possible response outputs of a fully functional p53 pathway is the induction of apoptosis generated by DNA damage. For apoptosis induction by radiation in certain tissues, a cascade of signaling is generated through pro-apoptotic proteins, such as response mediated by ATM protein (Bakkenist and Kastan, 2003). ATM leads to p53 stabilization, modifying the interaction capabilities with Mdm2 (El-Deiry, 1998). With the activation of p53, its degradation is limited, and p53 levels increase (Oliner et al., 1993). Downstream to ATM/p53 activation, an apoptosis program is carried out by a set of proteins, such as Bim, Puma, Bid, Bmf, Bad, Bik, Noxa, and Hrk, whereby Puma and Noxa can be directly regulated with p53 overexpression by gamma-radiation (Villunger et al., 2003; Chen et al., 2005). This set of proteins can bind and block survival proteins such as Bcl-2, which release death effectors like Bax and Bak. These effectors can lead to a change in the permeability of the outer mitochondria membrane. Furthermore, they can participate in the cell dismantling coupled with caspases (Green and Kroemer, 2004). There are other possible outcomes for the p53 activation pathway and p53 independent responses to DNA damage by gamma-radiation, which can lead the cell to cell cycle arrest, initiate DNA repair, or perform senescence. Experimental evidence indicates that the cellular level of p53 can dictate the response of the cell, such that lower levels of p53 result in arrest, whereas higher level results in apoptosis (Chen et al., 1996; Purvis et al., 2012).

#### 4.1.4. p53-Mdm2 Network With Nutlin-3

Just as p14ARF can stabilize p53 by antagonizing Mdm2 effects (Weber et al., 1999), if we consider molecules or treatments that can function as Mdm2 inhibitors, the small molecule Nutlin-3, is a compound described that binds in the p53-binding pocket within Mdm2, inhibiting its interaction with p53, and preventing p53 tagging for proteasome-mediated degradation (Vassilev et al., 2004; Yee-Lin et al., 2018).

Nutlin-3 is considered cytotoxic in specific wild-type p53 cancer cells (Vassilev et al., 2004; Arya et al., 2010; Yee-Lin et al., 2018), and therefore, p53 executes p53-dependent genes transactivation. Unlike in p53 mutated cell lines, transactivation is defective, and induced Mdm2 expression is impaired (Kamijo et al., 1998). It is essential to consider TP53 mutational status, other p53-dependent responses, and the Nutlin 3 activity in a dose and time-dependent manner (Arya et al., 2010), to predict the effect of Nutlin 3 in Mdm2 binding interactions.

### 4.2. Behaviors Induced by the Pinning Control Technique

#### 4.2.1. Case 1: Restoration of an Oscillatory Pattern Under Gamma-Radiation

The oscillatory pattern behavior (Geva-Zatorsky et al., 2006; Wee et al., 2009; Batchelor et al., 2011) induced by means of the pinning control technique is illustrated in Figure 6, with the purpose of restoring normal network behavior in presence of oncogenic overexpressed Mdm2; this overexpression avoids normal regulatory activities due to a suppressed wild-type p53. The pinning control technique (10) is located at the production of p14ARF node with *K*_*i*_ = 100, which allows to regenerate an oscillatory pattern and guarantees that p14ARF production *k*_*a*_*u*_1_ achieves a range between 1.6 and 9.5 *proteins*/*s*. Enhanced p14ARF production induces a decrease in Mdm2_*nuclear*_ which in turn increased p53 levels. This approach can help to analyze multiple interaction mechanisms, to induce different cell reprogramming responses. Therefore, the proposed approach motivates future researches on the interdependencies of cellular networks and new ways for treatment designs in tumor suppressor networks.

#### 4.2.2. Case 2: Achievement of an Increased p53 Level Expression

The increased expression of p53 levels by means of the pinning control technique is achieved as shown in Figure 7. Such technique generates the desired behavior of p53 progressive accumulation, assuming that this behavior has a post-translational activation mediated by ATM, which could generate the activation of downstream proteins, contributing to the activation of apoptosis or cell cycle arrest (El-Deiry, 1998; Wagner et al., 2005; Geva-Zatorsky et al., 2006; Wee et al., 2009). The pinning control technique (10) is located at the production of p14ARF node with *K*_*i*_ = 5, which yields an increased p53 level expression and guarantees that p14ARF production *k*_*a*_*u*_1_ achieves a range between 9.6 − 50*proteins*/*s*. p14ARF is moved from the nucleolus to nucleoplasm in response to DNA damage, where Mdm2_*nuclear*_-p14ARF complex promotes p53 tumor suppressor activity. For this case, it is possible to generate p53 accumulation, which is assumed to be activated by a mechanism linked to post-radiation activation of the ATM protein and p53-dependent induction of downstream proteins (Bakkenist and Kastan, 2003; Villunger et al., 2003; Pauklin et al., 2005). With the proposed pinning control law, it is possible to produce scenarios with different physiological or pathological responses of the p53-Mdm2 network.

For the two cases discussed above, the proposed pinning control strategy for the p53-Mdm2 network dynamics is applied on the p14ARF node based on sensitivity analysis as follows: In the first case, oscillatory pattern activity is achieved; in the second case p53 increased expression and accumulation are obtained. By means of the sensitivity analysis in *K*_*a*_ with respect to *p*53 and Mdm2_*nuclear*_, it can be established that with *K*_*a*_ low values, the network does not reach the desired behavior. However, for the adequate *K*_*a*_ values mentioned above, the network recovers its oscillatory pattern behavior, or an increase in p14ARF production leads causing a consistently increase p53 level. The proposed pinning control strategy as explained suppresses Mdm2 prooncogenic behavior and allows functional recovery of p53 physiological response.

#### 4.2.3. p14ARF as Pinned Node for p53-Mdm2 Network Control

In this section, we explain the importance of p14ARF as pinned node for the p53-Mdm2 network. Pinned node p14ARF regulates p53 by promoting Mdm2 degradation, preventing the Mdm2-mediated p53 degradation (Kamijo et al., 1998; Zhang et al., 1998; Weber et al., 1999). Experimental evidence indicates that p14ARF can even induce apoptosis through the Bax protein independent of p53 (Suzuki et al., 2003) and activate p53 through phosphorylation in oncogenic damage (Ito et al., 2001). It has also been reported that p14ARF helps to regulate the expression of Rad51, a protein involved in DNA repair; such regulation is explained by the activation of the ATM/ATR pathway, which is activated in response to DNA DSBs damage induced by ionizing radiation (Pauklin et al., 2005). In an *in vitro* approach (Itoshima et al., 2000), esophageal cancer cells were transfected with a plasmid designed to rise ARF expression (exogenous), with the subsequent reduction of endogenous levels of Mdm2 and induced p53 accumulation. Also, Itoshima et al. (2000), observed that mutant form of p53 was also stabilized by ectopic ARF increased expression, consistent with another report where p53 mutated can also be bonded to Mdm2 (Haupt et al., 1997); however, mutant-p53 transcriptional activity is obliterated and the existence of an intact autoregulatory loop between ARF, Mdm2, and p53 is needed to observe full regulatory pathway responses, particularly, a response that leads to apoptosis in cancer cells.

Interestingly, according to a model of overexpression of p14ARF in glioblastoma and astrocytoma cells, p14ARF controls neovascularization, through upregulation of metalloproteinase-3 inhibitor (TIMP3) in a p53-independent signaling pathway, which suppresses angiogenesis (Zerrouqi et al., 2012). Moreover, the upregulation of P14ARF could generate activation or interaction with other factors such as Sp1, c-Jun, AP-1, and JNK that cooperate to prevent tumor-induced microthrombosis through activation of the anticoagulant factor TFPI-2. p14ARF also enhances the transcriptional activity of Sp1, using the interaction of Mdm2 with Sp1. If Mdm2 is inhibited, Sp1 activity can be enhanced and induce tumor suppressor effects independent of p53-mediated signaling, as stated by Zerrouqi et al. (2014).

As future work, it would be essential to generate a new model that includes these and other interactions (disruption of mutant p53 stabilization, p53 post-translational modifications, external inhibitors, or altered proteasome system within the network), which would allow applying these control techniques and other types of analysis to others cell signaling pathways, to computationally model and stir the dynamics of a gene regulatory network to the desired state mainly to reproduce the coordinated fluctuation behavior of core components in p53 network.

Finally, experimental validation for the p53-Mdm2 network regulated by p14ARF as pinned node is not possible to implement due to lack of a biosensor measure the activity in the nucleus and cytoplasm at the cell. Our group is currently developing such a sensor.

## Data Availability Statement

The datasets generated for this study are available on request to the corresponding author.

## Author Contributions

OS, CV, ES, and GC initiated research and provided knowledge about strategies of control in the methods section. AG-S and OR-J proposed the description model and discussion. EH-V and AA discussed simulations and prepared figures. All authors designed the research, analyzed data, prepared, wrote, and reviewed the paper.

## Conflict of Interest

The authors declare that the research was conducted in the absence of any commercial or financial relationships that could be construed as a potential conflict of interest.
